# High‐Rate Cross‐Channel Entanglement Swapping Between Independent On‐Chip Sources

**DOI:** 10.1002/advs.202518802

**Published:** 2025-12-05

**Authors:** Haoyang Wang, Huihong Yuan, Qiang Zeng, Lai Zhou, Haiqiang Ma, Zhiliang Yuan

**Affiliations:** ^1^ Beijing Academy of Quantum Information Sciences Beijing 100193 China; ^2^ School of Physical Science and Technology Beijing University of Posts and Telecommunications Beijing 100876 China; ^3^ State Key Laboratory of Information Photonics and Optical Communications Beijing University of Posts and Telecommunications Beijing 100876 China

**Keywords:** cross‐channel, entanglement swapping, integrated quantum chip, silicon on insulate, time‐bin

## Abstract

The emerging integrated quantum photonics is pushing forward the establishment of quantum networks. To interconnect individual chip‐based quantum sources, high‐performance entanglement swapping is indispensable. Here, using low‐loss silicon chips with waveguide structure and frequency‐offset pumps, high‐rate cross‐channel entanglement swapping is demonstrated. A record‐high swapping rate of 207 counts per hour is obtained, and the measured swapping visibilities between each of two pairs of channels exceed 90%. The proposed cross‐channel architecture allows dynamic channel switching by adjusting pump wavelengths, enabling flexible user pairing in quantum networks. This work makes a concrete benchmark for chip‐based entanglement swapping and provides a viable solution for real‐world quantum communication.

## Graphical abstract

To interconnect individual chip‐based quantum sources, high‐performance entanglement swapping is indispensable. Here, using low‐loss silicon chips with waveguide structure and frequency‐offset pumps, this work demonstrates high‐rate cross‐channel entanglement swapping. A record‐high swapping rate of 207 counts per hour is obtained, and the measured swapping visibilities between each of two pairs of channels exceed 90%. The proposed cross‐channel architecture allows dynamic channel switching by adjusting pump wavelengths, enabling flexible user pairing in quantum networks.

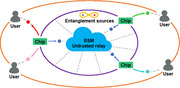

## 1 Introduction

Entanglement has emerged as a fundamental resource for quantum information processing.^[^ ^1^, ^2^, ^3^, ^4^ ^]^ To extend its utility toward building large‐scale quantum networks, entanglement swapping becomes essential: it establishes entanglement between distant parties that never directly interact by means of Bell‐state measurements (BSM) on photons received from remote subsystems at an intermediate node.^[^ ^5^ ^]^ This essential protocol enables quantum repeaters^[^ ^6^, ^7^ ^]^ and relays,^[^ ^8^, ^9^, ^10^ ^]^ mitigating the limit of transmission distance imposed by quantum channel loss. Moreover, entanglement swapping between adjacent nodes facilitates interconnections among diverse quantum devices, enabling qubit information exchange across networks and laying the groundwork for complex applications such as distributed quantum computing and quantum sensing.^[^ ^11^, ^12^, ^13^ ^]^

Although originally proposed with independent sources,^[^ ^5^ ^]^ early demonstrations of entanglement swapping employed duplicate sources pumped by a common laser,^[^ ^14^, ^15^, ^16^ ^]^ and only later was the scheme realized with truly independent sources.^[^ ^17^, ^18^, ^19^, ^20^ ^]^ Sources based on bulk optical materials can offer high fidelity^[^ ^18^ ^]^ while those employing dispersion‐shifted fibers have demonstrated transmission over 100 km.^[^ ^19^ ^]^ With the advance of integrated photonic circuits, on‐chip photon‐pair sources—such as resonators and waveguides—are gaining prominence due to their miniaturization, compatibility, and cost‐effectiveness.^[^ ^21^ ^]^

**Figure 1:**
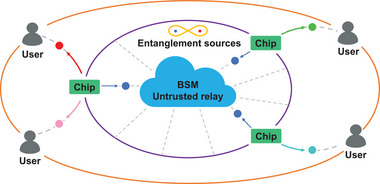
Schematic of cross‐channel entanglement swapping. Each on‐chip entanglement source distributes signal and idler photons to users and a central untrusted relay, respectively. The pump wavelength is chosen so that the idler photons are indistinguishable and the signal photons correspond to end‐users' channel. In particular, the signal channels can be switched by tuning the pump wavelength as shown in the single chip on the left. The relay performs Bell‐state measurements to entangle remote signal photons at users on demand.

Independent sources further enable novel relay architectures for quantum networks. As illustrated in **Figure** **1**, distant on‐chip twin‐photon emitters are excited individually by pumps with frequency offset, distributing identical idler photons for entanglement swapping and distinct signal photons to end‐users. The cross‐channel distribution is realized either through tuning the pump wavelength at a single chip, or using respective pumps at individual chips. This cross‐channel topology provides flexibility in connecting any pair of users in the network—communication channels can be swiftly switched by adjusting the wavelength of pump. Such an architecture not only maximizes relay throughput but also enables scalable and reconfigurable user pairing across the network. Moreover, our proposal naturally can adapt to high‐dimensional and multiplexed entanglement sources.^[^ ^22^ ^]^

Recent progress has enabled entanglement swapping between independent chip‐based photon‐pair sources, first realized using microring resonators.^[^ ^20^ ^]^ Resonant cavities, in particular, generate entangled photons with high spectral purity,^[^ ^23^, ^24^ ^]^ enabling high‐visibility two‐photon interference. However, such cavity structures are temperature‐sensitive and vulnerable to environmental perturbations. Moreover, the strong optical confinement responsible for their spectral purity also reduces heralding efficiency^[^ ^25^ ^]^ and compromises the simultaneity of photon‐pair emission,^[^ ^26^, ^27^ ^]^ placing an intrinsic upper bound on the entanglement‐swapping rate.

In contrast, spiral waveguides avoid temporal uncertainty during entanglement generation^[^ ^28^ ^]^ thus allowing high‐resolution temporal measurement, but the challenges are relatively high on‐chip transmission and spectral filtering cost.^[^ ^29^ ^]^ In this work, we develop low‐loss waveguide chips based on silicon‐on‐insulator (SOI) technology to construct high‐performance entanglement sources. The photon‐pairs sources are independently stimulated by high‐repetition‐rate pulsed pumps up to 2.5 GHz, with a 50 GHz center‐frequency offset. Using narrow‐band filtering, we demonstrate Hong‐Ou‐Mandel (HOM) interference and entanglement swapping using time‐bin encoding, achieving both high rate and high visibility among two pairs of signal channels. Our cross‐channel swapping platform is robust to disturbances, and supports high‐rate encoding and dynamic channel switching, making it competitive for real‐world quantum communication.

## 2 Experimental Section

**Figure 2:**
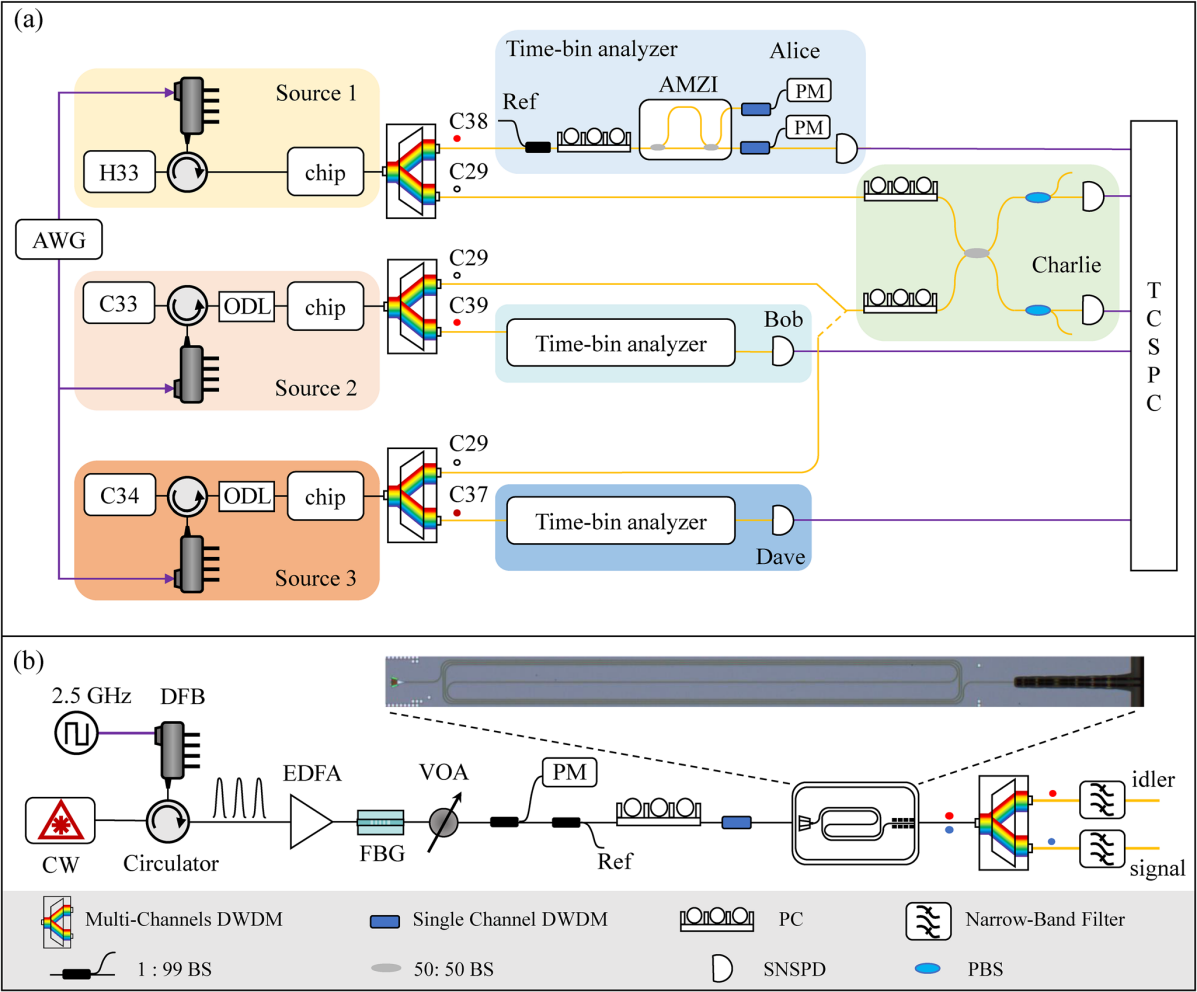
Experimental schematic. a) Cross‐channel entanglement swapping experimental realization. Two pairs of independent entanglement sources are generated by SFWM process in waveguides, which are stimulated by pulsed pump with a repetition rate of 2.5 GHz. Pulse sequences are synchronized by an arbitrary waveform generator (AWG). The idler photons (C29) are sent to Charlie for Bell state measurement, and signal photons (C37, C38, and C39) are distributed to Alice, Bob and Dave respectively for entanglement verification. Coincidence is measured by a time‐correlated single photon counting module (TCSPC). Setups: ODL, optical delay line; AMZI, asymmetric Mach‐Zehnder interferometer; DWDM, dense wavelength‐division multiplexer; BS, beam splitter; SNSPD, superconducting nanowire single‐photon detector. PC, polarization controller. PBS, polarization beam splitter. b) Time‐bin entanglement source. Setups: CW, continuous‐wave laser; DFB, distributed feedback laser; EDFA, erbium‐doped fiber amplifier; FBG, fiber Bragger grating; VOA, variable optical attenuator; PM, power meter. The inset is the enlarger photo image of the silicon chip.

For demonstrating cross‐channel entanglement swapping, three independent entangled photon‐pair sources were employed in the setup shown in **Figure** **2**a. Figure 2b depicts the schematic of the source. A distributed feedback (DFB) laser was driven by a 2.5 GHz square signal from an arbitrary waveform generator (AWG), producing optical pulses. Note that every two adjacent pulses form a time‐bin qubit, preserving the phase information. To enable phase coherence as well as improving intensity stability, a continuous‐wave (CW) laser was used to seed the DFB laser via an optical circulator^[^ ^30^, ^31^ ^]^ (see Section S1, Supporting Information for details). The generated pulses were amplified by an erbium‐doped fiber amplifier (EDFA) and filtered by a followed fiber Bragg grating (FBG) with a 0.2 nm full‐width‐at‐half‐maximum (FWHM) and an side‐band‐isolation of about 60 dB. The average power of the pump pulses were actively stabilized by a variable optical attenuation (VOA), achieved by feeding 1% of its intensity through a 1:99 beam splitter (BS) to an optical power meter (PM).

The pump pulses were aligned to the quasi‐transverse electric (TE) mode of the waveguide using a polarization controller (PC), then filtered by a single‐channel dense wavelength‐division multiplexer (DWDM) to suppress Raman scattering noise, and finally injected into the spiral waveguide to generate entangled photon pairs (see Appendix A for fabrication details). The interaction between the pump and the waveguide induced spontaneous four‐wave mixing (SFWM) process, in which the annihilation of two pump photons created a signal photon and an idler photon.

Due to energy conservation, the signal (ω_*s*_) and idler (ω_*i*_) photons were symmetrically distributed around the pump frequency (ω_*p*_). The generated photon pairs were separated by a multi‐channel DWDM with a 0.6 nm FWHM and a side‐band isolation of about 80 dB. Subsequent narrow‐band filters with a 0.05 nm bandwidth–approximately one quarter of the pump spectral width–further purify the photons, ensuring a spectral purity exceeding 99.9%.^[^ ^29^ ^]^ The spectral characteristics of the DWDM and narrow‐band filter are provided in Section S2 (Supporting Information).

Source 1 was pumped by the International Telecommunication Union (ITU) channel wavelength H33 (1550.52 nm), producing signal photons at C38 (1546.92 nm) sent to Alice for time‐bin analysis and idler photons at C29 (1554.13 nm) to Charlie for BSM. Similarly, source 2 was pumped at C34, with Bob receiving the corresponding signal photons at C39 (1546.12 nm). For pairing between Alice and Dave, source 1 remains the same, while source 3 was pumped at C33 (1550.92 nm), generating signal photons at C37 (1547.72 nm) that were sent to Dave. In the proof‐of‐principle demonstration, Bob and Dave share a single photon detector, and their idler channels were connected to Charlie in an alternating manner.

For entanglement swapping, the idler photons from each participating source at C29 were directed to Charlie for Hong–Ou–Mandel (HOM) interference. To ensure simultaneous arrival at Charlie's 50/50 beamsplitter, an optical delay line (ODL) was inserted in sources 2 and 3 to compensate for path‐length differences. The signal photons were analyzed using two home‐built asymmetric Mach–Zehnder interferometers (AMZIs) with a differential delay of 400 ps, matched to the 2.5 GHz pump clock that drives each waveguide chip. Photons were detected by superconducting nanowire single‐photon detectors (SNSPDs) with a detection efficiency of 85%, a dark count rate of 150 Hz, and a timing jitter of 40 ps. The detector signals were then sent to a time‐correlated single‐photon counting (TCSPC) module for coincidence measurements, which had a jitter of 8 ps.

Before swapping taking place, the entanglement state of each individual source can be written as [31]:

(1) $${\left|\Theta \right\rangle }_{k}=\frac{1}{\sqrt{2}}\left({\left|E\right\rangle }_{s}{\left|E\right\rangle }_{i}+e^{i\theta_{k}}{\left|L\right\rangle }_{s}{\left|L\right\rangle }_{i}\right)$$

where |E〉(|L〉) refers to early(late) time‐bin, and θ_*k*_, *k* ∈ {1, 2, 3} is the relative phase between two time‐bins, which is defined by the respective pump wavelength. The swapping operation projects the combined four‐photon state onto the four Bell states as (exemplified by sources 1 and 2)

(2) $$\begin{array}{cc} {\left|\Theta \right\rangle }_{1}{\left|\Theta \right\rangle }_{2}= & \frac{1}{2\sqrt{2}}\left[\left({\left|E\right\rangle }_{1s}{\left|E\right\rangle }_{2s}+e^{i(\theta_{1}+\theta_{2})}{\left|L\right\rangle }_{1s}{\left|L\right\rangle }_{2s}\right){|\Phi^{+}\rangle }_{i}\right.\\ & \left.+\left({\left|E\right\rangle }_{1s}{\left|E\right\rangle }_{2s}-e^{i(\theta_{1}+\theta_{2})}{\left|L\right\rangle }_{1s}{\left|L\right\rangle }_{2s}\right){|\Phi^{-}\rangle }_{i}\right.\\ & \left.+\left(e^{i\theta_{2}}{\left|E\right\rangle }_{1s}{\left|L\right\rangle }_{2s}+e^{i\theta_{1}}{\left|L\right\rangle }_{1s}{\left|E\right\rangle }_{2s}\right){|\Psi^{+}\rangle }_{i}\right.\\ & \left.+\left(e^{i\theta_{2}}{\left|E\right\rangle }_{1s}{\left|L\right\rangle }_{2s}-e^{i\theta_{1}}{\left|L\right\rangle }_{1s}{\left|E\right\rangle }_{2s}\right){|\Psi^{-}\rangle }_{i}\right] \end{array}$$

where the four Bell states are defined as ${|\Phi^{\pm }\rangle }_{i}=\frac{1}{\sqrt{2}}\left({\left|E\right\rangle }_{1i}{\left|E\right\rangle }_{2i}\pm {\left|L\right\rangle }_{1i}{\left|L\right\rangle }_{2i}\right)$, and ${|\Psi^{\pm }\rangle }_{i}=\frac{1}{\sqrt{2}}\left({\left|E\right\rangle }_{1i}{\left|L\right\rangle }_{2i}\pm {\left|L\right\rangle }_{1i}{\left|E\right\rangle }_{2i}\right)$. Thus, successful Hong–Ou–Mandel interference at Charlie effectively projects the idler photons into a Bell state and, as a result, heralds entanglement between the remote signal photons. Because the employed SNSPDs were not photon‐number‐resolving and have a dead time of ≈80 ns, much longer than the 400 ps time‐bin separation, Charlie cannot discriminate Bell states, where both idler photons exit from the same port of the HOM beam splitter, namely |Φ^±^〉_*i*_ and |Ψ^+^〉_*i*_.^[^ ^19^, ^20^ ^]^ The only measurable Bell state is |Ψ^−^〉_*i*_, whose detection projects the signal photons onto

(3) $$|\Theta_{s}\rangle =\frac{1}{\sqrt{2}}\left({\left|E\right\rangle }_{1s}{\left|L\right\rangle }_{2s}-e^{i(\theta_{1}-\theta_{2})}{\left|L\right\rangle }_{1s}{\left|E\right\rangle }_{2s}\right)$$

In the projected state, two signal photons were entangled in the post‐selected manner.

To witness that the entanglement was swapped to the remote photons, each of the two photons is routed to a 400‐ps‐delay AMZI and detected by an SNSPD. Detection events were time‐tagged by a TCSPC module (Swabian Time Tagger Ultra, 1‐ps resolution). Fourfold coincidence events are sifted, where the twofold coincidence of the idler photons heralds the remote entanglement of the signal photons. To observe the entanglement interference fringes, the relative phase of the two AMZIs deployed at Alice and Bob/Dave was tuned via temperature control. Phase drifts in each local AMZI were actively stabilized by locking the reference phase to the pump pulses via a TEC and PID feedback system.

## 3 Results

**Figure 3:**
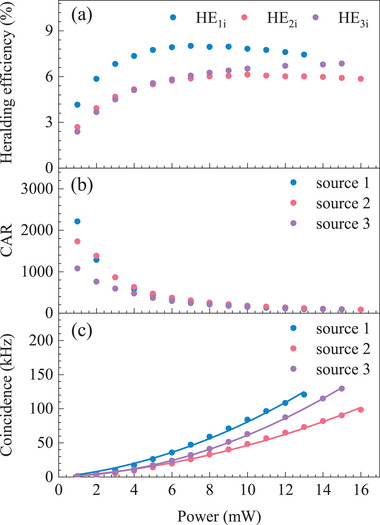
Photon pair source performance: a) Heralding efficiency (HE) as a function of the pump power. b) CAR as a function of the pump power. c) Photon‐pair source coincidence as a function of the pump power. The experimental data are fitted with *aP* ^2^ + *bP*, where *P* is the pump power, *a* is the quadratic SFWM term, and *b* is the noise photons term.

We first characterize twofold correlations of the individual photon‐pair sources. The heralding efficiencies exemplified by signal channels are presented in **Figure** **3**a, which characterizes the overall photon loss from sources to users. The channel collection efficiency is calibrated using classical light, including chip transmission loss of 1.5 dB, chip‐to‐fiber coupling loss of 1.5 dB, DWDM insertion loss of 1.3 dB, narrow‐band filter insertion loss of 0.6 dB, fiber link loss of 0.1 dB, and detector loss of 0.7 dB, thus of 5.7 dB in total. We attribute the gap between heralding and collection efficiency to the narrow‐band filtering inefficiency.^[^ ^29^ ^]^

The coincidence and coincidence‐to‐accidental ratio (CAR) versus pump power are reported in Figure 3b,c, respectively. The coincidence rate scales quadratically with the pump power, which is in accordance with the SFWM process in the waveguide.

We then conduct HOM interference for the two pairs of sources respectively with a coincidence window of 400 ps. It is desirable to achieve both high fourfold coincidence rate and high HOM visibility, but theory indicates a trade‐off. In specific, the visibility $V_{\text{HOM}}\approx \frac{\text{CAR}+7}{\text{CAR}+11}$,^[^ ^32^ ^]^ assuming the noises are sufficiently suppressed thus $\text{CAR}\approx \frac{1}{\mu }+1$,^[^ ^33^ ^]^ where µ is the average photon number. On the other hand, the fourfold coincidence rate is $C_{\text{HOM}}=\frac{\eta_{1s}\eta_{1i}\eta_{2s}\eta_{2i}R}{2{\left(\text{CAR}-1\right)}^{2}}$, where *R* is the repetition rate of pulses, and η_*s*1_, η_*i*1_, η_*s*2_, η_*i*2_ are the heralding efficiency of signal and idler photons from the two sources.

**Figure 4:**
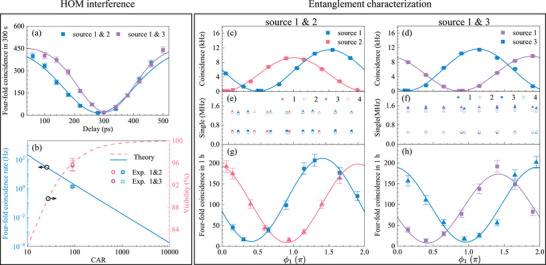
Hong–Ou–Mandel (HOM) interference and entanglement swapping results. a) HOM dip: fourfold coincidence counts versus time delay. b) HOM rate and visibility versus CAR. Blue solid line: theoretical HOM rate estimated based on measured heralding efficiency; Red dashed line: ideal HOM visibility estimated based on measured average CAR; Pink and violet dots: measured HOM rate and visibility, respectively. c,d) Time‐bin entanglement of sources 1 and 2, and 1 and 3. Coincidence rates of the entanglement sources between the signal and idler as a function of the relative phase of AMZI1 (ϕ_1_) placed at Alice's. e,f) Single count rate of four SNSPDs when measuring the swapping rate. g,h) Entanglement swapping between sources 1 and 2 and sources 1 and 3. Fourfold coincidence counts in one hour as a function of ϕ_1_. Bob's phase is fixed at 0.35~π and 0.85~π while Alice's phase is swept, generating two fringes. The visibilities of the fitted curve are 91.4% ± 2.8% (red) and 89.5% ± 3.0% (blue). For sources 1 and 3, the visibilities are 91.7% ± 3.7% (blue) and 90.4% ± 2.6% (purple).

To achieve both high HOM interference visibility and a high fourfold coincidence rate, we set the photon pair generation rate of each source to be ≈0.011. To this end, we control the pump power so that the photon‐pair sources have similar CAR and coincidence rate. In specific, source 1–3 are pumped at 13, 16, and 15 mW, yielding coincidence rates around 100 kHz with coincidence‐to‐accidental ratios (CARs) of 97, 84, and 85, respectively. The pump powers differ by ⩽0.7 dB among the three sources, which is within the estimation as the chip‐to‐fiber coupling loss is ⩽ 0.2 dB per facet and the wavelength‐dependent insertion loss of the DWDM is ⩽0.4 dB. Using an average CAR of 90 between source 1 and source 2 (3), we estimate the theoretical fourfold coincidence rate to be 2.2 Hz, and the interference visibility to be 96.0%. The fourfold coincidence histogram as a function of the delay between two idler photons is shown in **Figure** **4**a. The measured HOM dip visibility between source 1 and source 2 is 95.7% ± 1.1% with a maximum fourfold coincidence rate of 1.33 Hz, and the HOM visibility between sources 1 and 3 is 95.5% ± 1.0% with a maximum fourfold coincidence rate of 1.46 Hz, which are in good agreement with the theoretical value, as depicted in Figure 4b. We attribute the minor gap in fourfold coincidence rate between experiment and theory to the extra losses introduced by the fiber components within the interferometer at Charlie, which is approximately of 1 dB for each path.

We then characterize the entanglement visibility of the individual sources involved in entanglement swapping experiments. In this case, we deploy two time‐bin analyzers in the signal and idler path, adjust the relative phase, and monitor the twofold coincidence rate. The results are shown in Figure 4c, d, and the raw visibilities are 97.8% and 98.4% for sources 1 and 2, respectively. For sources 1 and 3, the raw visibilities are 97.3% and 98.5%, respectively.

Finally, we present the results of entanglement swapping experiments in Figure 4e–h). Figure 4e,f shows the single count rates of the four SNSPDs when we measure the fourfold coincidence counts, demonstrating the stability of the chip‐to‐fiber coupling. The fourfold coincidence counts show a clear interference fringe, as shown in Figure 4g,h, indicating the entanglement between the remote signal photons. The average visibility of the fitted curves between sources 1 and 2 is 90.4% ± 2.1%, and the maximum swapping rate is 207 counts per hour. For sources 1 and 3, the average visibility is 91.1% ± 2.6% and swapping rate 202 per hour.

## 4 Discussion

**Table** **1** summarizes previous demonstrations of chip‐based entanglement swapping, including both duplicate and independent sources. Our work achieves improvements across all key metrics, enabled by advanced silicon chip design and high pump repetition rates. However, achieving a swapping rate above 1 Hz—a key milestone—still remains challenging. There are several ways to further improve the rate. First, narrow‐band filtering ensured high spectral purity but introduced excess loss, reducing heralding efficiency to about a quarter of actual transmittance. We note that recent progress in spectral engineering of waveguides^[^ ^37^, ^38^, ^39^ ^]^ may enable high purity without filtering, offering a pathway toward higher rates.

**Table 1:** Comparing HOM interference and entanglement swapping parameters in integrated telecom photon‐pair sources.

Refs.	Source	Pump	*V*_HOM_	HOM rate	*V*_Swap._	Swap. rate
		(ITU channel)		Hz		cph
**This work**	Si waveguide	2.5 GHz @H33/C34	95.7% ± 1.1%	1.33	90.4% ± 2.1%	207
		@H33/C33	95.5% ± 1.0%	1.46	91.1% ± 2.6%	202
[20]	Si_3_N_4_ microring	CW @C25/C27	93.2% ± 1.6%	0.13	89.7% ± 2.4%	33
[34]	Si waveguide	50 MHz @C27/C28	88% ± 8%	0.006	—	—
[35]	Si waveguide^1^	500 MHz @C34	73%	0.011	—	—
[36]	PPLN waveguide	76 MHz @768 nm	93% ± 3%	0.003	—	—
[17]	PPLN waveguide	CW @780 nm	77%	0.0003	63% ± 2%	5

1 Duplicate sources

Second, the filtering cost on the reference light in the AMZI (about 2 dB each) reduced the swapping rate. In our experiments, the reference light is co‐propagating with the signal photons entering the interferometer from the same input. A filter is used to separate the reference light but introduces additional loss. We note that the alternative counter‐propagating reference light can avoid this loss but requires a high return isolation.

Third, temporal jitter of the measurement devices limited the usable coincidence window and thus the coincidence rate. The 2.5 GHz pulses corresponds to a 400 ps time interval. To avoid cross‐talk between adjacent time‐bins and thus improve the interference visibility, we reduce the coincidence window width to 300 ps (see Section S3, Supporting Information for details). Future work may employ shorter pulses and lower‐jitter detectors to improve both rate and visibility.

The deployment of proposed platform over long‐haul fibers (over 100 km) is confronted by several key obstacles that impact both the signal rate and quantum‐state integrity. The major issue is that the channel loss drastically reduces the fourfold coincidence rate—projected to be a mere 0.014 Hz at 100 km—a limitation that may be partially addressed by deploying ultra‐low‐loss fibers. In addition, the fidelity of the quantum states could be compromised by dynamic polarization and phase drifts, demanding the implementation of robust, active calibration systems. Moreover, temporal dispersion introduces arrival‐time jitter, which blurs the precise timing correlations that are essential for high‐visibility quantum interference and the execution of distributed protocols.

## 5 Conclusion

In summary, we conceptualized the cross‐channel entanglement swapping architecture, based on which the communication channels in the networks can be efficiently exploited. We have demonstrated cross‐channel entanglement swapping between two pairs of independent chip‐based time‐bin entanglement sources, and each pair of pumps have a 50 GHz‐spacing. With 2.5 GHz pump pulses and high‐performance photon‐pair sources, we achieved swapping rates of 200 counts per hour and HOM rates exceeding 1 Hz, while maintaining >90% entanglement visibility and >95% HOM visibility. Our SOI waveguide sources are shown competitive in all key merits for entanglement swapping applications. Combining with the new cross‐channel architecture, our platform provides a promising solution for future distributed long‐distance quantum communication networks and potentially quantum computation.

## Conflict of Interest

The authors declare no conflict of interest.

## Supporting information

Supporting Information

## Figures and Tables

**Figure A.1:**
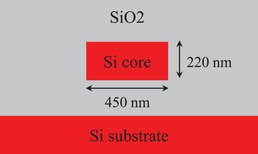
Schematic of the waveguide cross section.

## Data Availability

The data that support the findings of this study are available from the corresponding author upon reasonable request.

## Appendix A Waveguide Chip Fabrication

The cross‐section schematic of our SOI chip is presented in **Figure** A.1. Fabricated on a Si wafer, the spiral waveguide is designed for TE mode transmission and has a cross‐section dimension of 450 × 220 nm. To minimize transmission losses, each waveguide bend was designed to be no less than 22 µm. Additionally, a fine annealing process was performed during fabrication to reduce the waveguide sidewall roughness after dry etching the silicon layer during fabrication. The grating coupler as input is for flexibility and suspended edge coupler as output is to minimize the output coupling loss of the generated photon pairs.^[^ ^40^ ^]^ The total loss on chip of our sources is 7.5 dB on average, including grating coupling loss 4.5 dB, waveguide transmission loss 1.5 dB, and edge coupling loss 1.5 dB.
