# Supplementary material for: High‐Rate Cross‐Channel Entanglement Swapping Between Independent On‐Chip Sources
Source: Adv Sci (Weinh). 2025 Dec 5;13(9):e18802. doi: 10.1002/advs.202518802 (PMC12903983; doi:10.1002/advs.202518802)
Supplement: Supplementary file 1 — Supporting Information [file ADVS-13-e18802-s001.pdf]

Supplementary Material: High-rate cross-channel entanglement swapping between independent on-chip sources

Haoyang Wang,[1,2,3] Huihong Yuan,[1] Qiang Zeng,[1,*] Lai Zhou,[1] Haiqiang Ma,[2,3] and Zhiliang Yuan[1,†]

[1]*Beijing Academy of Quantum Information Sciences, Beijing 100193, China*
[2]*School of Physical Science and Technology, Beijing University of Posts and Telecommunications, Beijing 100876, China*
[3]*State Key Laboratory of Information Photonics and Optical Communications, Beijing University of Posts and Telecommunications, Beijing 100876, China*

S1. Optical injection locking

We employ optical injection locking approach to ensure coherence among generated pump pulses. The experimental setup is illustrated in Fig. S1 (a), a CW tunable laser (Santec TSL-770, $\leq$60 kHz linewidth) is connected to a distributed feedback laser triggered by a 2.5 GHz microwave signal via an optical circulator with an injected power of 0.45 mW. The relative phase between the pulses generated by the injection locking set is determined by the injected CW light. The mechanism behind this phase stabilization can be outlined as follows: when the gain-switch is "off", the carrier density in the DFB is close to transparency, and thus the injected field can seed the cavity mode and impose its phase noise to the slave pulse. To compare phase variation with and without optical injection, we send the pulses passing through an AMZI with a 400-ps arm difference and monitor the power meter. We alter the initial phase by slightly adjust the wavelength of the CW laser to make a comparison to the case without injection locking.

As shown in Fig.S1 (b), the results reveals that under the optical injection condition, the phase between adjacent pulses is stabilized at 0.35 π rad with a standard deviation of 0.0025 π rad over ten hours. Without optical injection, the measured phase is invariant at 0.495 π rad with a standard deviation of 0.0003 π rad, which is reasonable because the pulses are totally incoherent and therefore the optical power is equally distributed to the two arms of the interferometer. The value is not an exact 0.5 π rad only due to the imbalanced losses of the two arms of the AMZI.

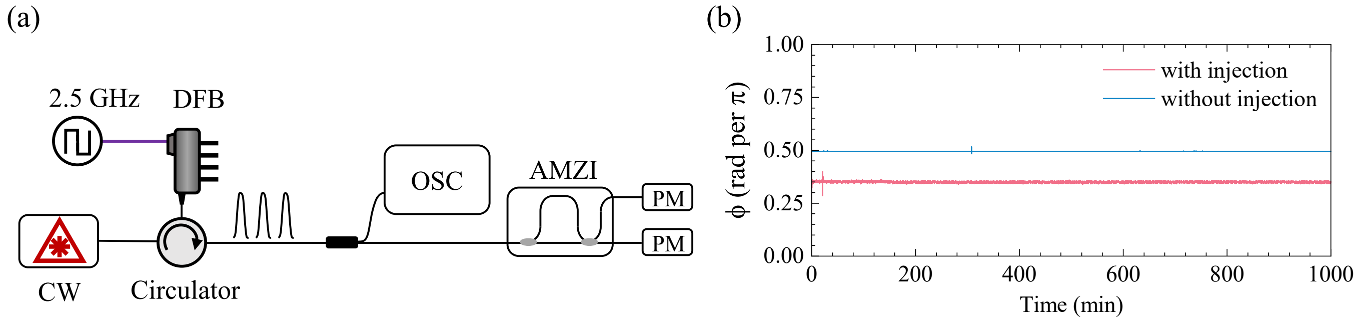

FIG. S1. (a) Schematic of the optical injected locking pulses (b) Phase monitoring for 1000 minutes. The red line represents the measurement of pulses with optical injection, while the blue line represents the results without optical injection

Additionally, we measured the pulse duration and timing jitter using an oscilloscope (33 GHz bandwidth) with and without injection by the CW laser; the results are shown in Table. S1.

TABLE S1. optical injection locking characteristics

CW	pulse width	time jitter
H33	40.9 ps	0.2 ps
C33	39.6 ps	2.0 ps
C34	38.8 ps	0.6 ps
w/o	32.8 ps	7.6 ps

S2. Filter system

The filtering system includes a multi-channel DWDM (Optizonetech) and narrow-band filters (QuantumCTek, GD311). Using a spectrum-scanning system, we measured their insertion loss, 3dB bandwidth, and isolation, with the results shown in Figure S2.

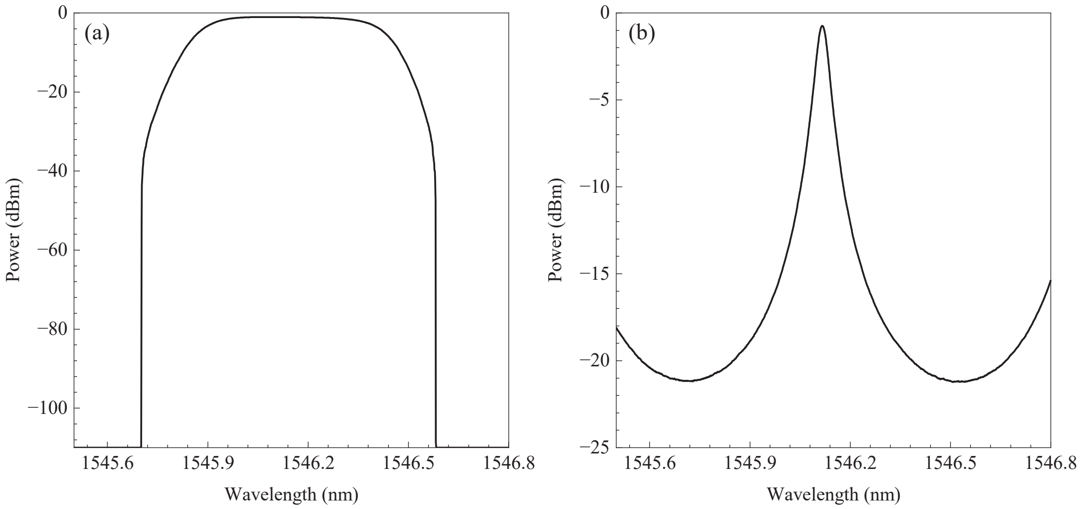

FIG. S2. (a) DWDM and (b) narrowband filter spectrum sweep test results.

S3. Time-bin entanglement

We measured the interference visibility of each time-bin entanglement source using the experimental setup shown in Fig. S3. A train of pump pulses with fixed phase is sent to a silicon waveguide chip, where the time-bin entanglement state $|\Theta\rangle_k = \frac{1}{\sqrt{2}}(|E\rangle_s|E\rangle_i + e^{i\theta_k}|L\rangle_s|L\rangle_i)$, is realized through a spontaneous four-wave mixing (SFWM) process. The generated signal photons and idler photons are filtered through a DWDM and narrowband filters before being transmitted to Alice and Bob's AMZI for analysis and measurement, respectively.

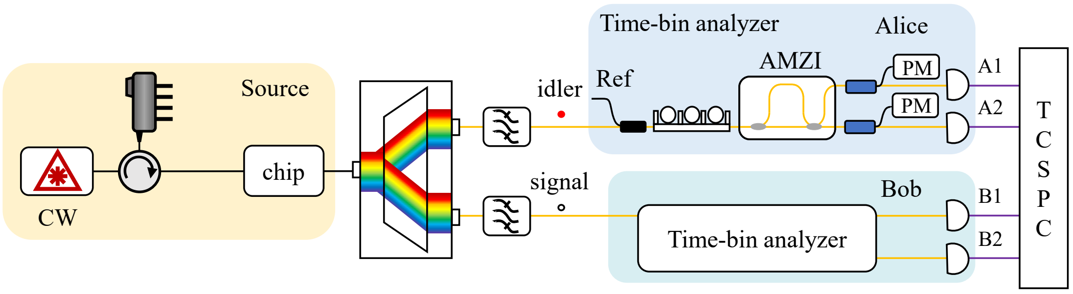

FIG. S3. Schematic of the time-bin entanglement measurement.

The path difference in the two arms of the AMZI imposes distinct time delays on the transmitted photons. This delay structure produces three peaks in the two-photon coincidence measurement (Fig. S4). The central peak originates from the indistinguishable cases in which the two photons propagate through either both short or both long arms simultaneously. This indistinguishability gives rise to quantum interference, causing the central peak's coincidence count to vary with the relative phase between the AMZI arms. The side peaks, however, correspond to distinguishable events where one photon travels through the long arm and the other through the short arm. Consequently, these peaks exhibit no interference effect.

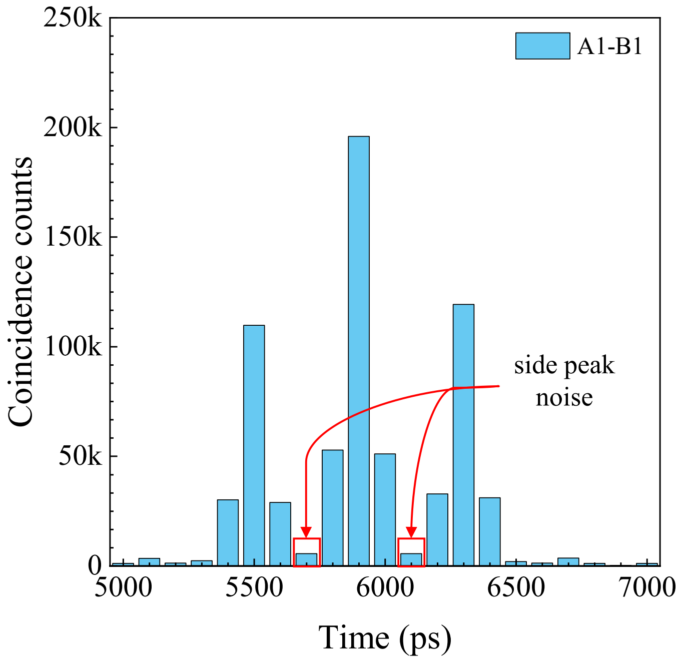

FIG. S4. Coincidence histogram of SNSPD A1 and B1.

As shown in Fig. S4, a 400 ps coincidence window unavoidably incorporates events from the side peaks, where no interference occurs. To ensure signal purity in the entanglement measurement and the subsequent entanglement swapping experiment, we therefore adopted a 300 ps coincidence window.

* zengqiang@baqis.ac.cn
† yuanzl@baqis.ac.cn
